# Quality Control of PSII: Behavior of PSII in the Highly Crowded Grana Thylakoids Under Excessive Light

**DOI:** 10.1093/pcp/pcu043

**Published:** 2014-03-26

**Authors:** Yasusi Yamamoto, Suguru Kai, Atsuki Ohnishi, Nodoka Tsumura, Tomomi Ishikawa, Haruka Hori, Noriko Morita, Yasuo Ishikawa

**Affiliations:** Graduate School of Natural Science and Technology, Okayama University, Okayama, 700-8530 Japan

**Keywords:** Light stress, Membrane crowdedness, Non-photochemical quenching, Photoinhibition, Photosystem II, Protein aggregation

## Abstract

The grana thylakoids of higher plant chloroplasts are crowded with PSII and the associated light-harvesting complexes (LHCIIs). They constitute supercomplexes, and often form semi-crystalline arrays in the grana. The crowded condition of the grana may be necessary for efficient trapping of excitation energy by LHCII under weak light, but it might hinder proper movement of LHCII necessary for reversible aggregation of LHCII in the energy-dependent quenching of Chl fluorescence under moderate high light. When the thylakoids are illuminated with extreme high light, the reaction center-binding D1 protein of PSII is photodamaged, and the damaged protein migrates to the grana margins for degradation and subsequent repair. In both moderate and extreme high-light conditions, fluidity of the thylakoid membrane is crucial. In this review, we first provide an overview of photoprotective processes, then discuss changes in membrane fluidity and mobility of the protein complexes in the grana under excessive light, which are closely associated with photoprotection of PSII. We hypothesize that reversible aggregation of LHCII, which is necessary to avoid light stress under moderate high light, and swift turnover of the photodamaged D1 protein under extreme high light are threatened by irreversible protein aggregation induced by reactive oxygen species in photochemical reactions.

## Introduction

Plants respond to long-term change in light conditions, such as seasonal or locational variation in light intensity, by acclimation, whereas short-term, ever-changing light conditions may sometimes cause excessive irradiation of plants and result in short-term light stress. In the latter instance, chloroplasts show unique responses to overcome the damaging effects of excessive illumination within a short period. In this review, we focus on the molecular and dynamic behavior of PSII and the thylakoid membranes of higher plant chloroplasts in response to short-term light stress.

**Fig. 1:**
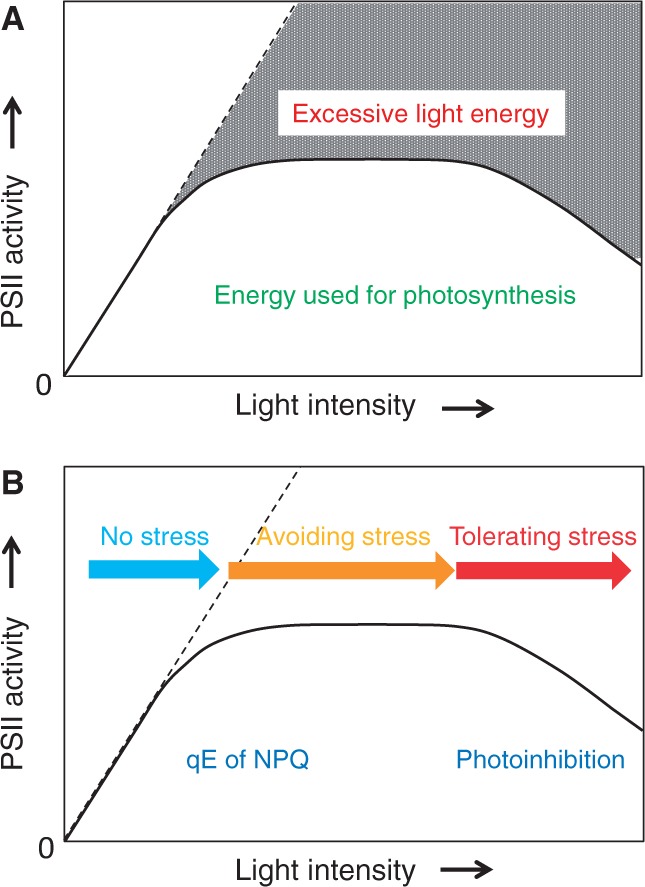
Light response curve for PSII activity. The solid line shows PSII activity at various light intensities, while the broken line represents the input energy, or PSII activity in the absence of any rate-limiting factors. (A) Energy usage in PSII. The gray shaded area represents excessive light energy. The area below the solid line corresponds to the energy used for photosynthesis. (B) Relationship between qE of NPQ and photoinhibition of PSII in the light–response curve. Under low light, PSII activity increases in proportion to the light intensity. No light stress is likely under this condition (indicated by the blue arrow). With increasing light intensity, PSII activity gradually decreases and reaches a plateau. Under these conditions, chloroplasts avoid light stress using the qE mechanism of NPQ (indicated by the orange arrow). The qE takes place through aggregation of LHCII activated by acidification of the thylakoid lumen and zeaxanthin formation by the xanthophyll cycle, and it acts to dissipate excessive light energy as heat. Under high-intensity light, photoinhibition prevails. PSII tolerates the severe light stress by stimulating damage and repair of the reaction center-binding D1 protein (indicated by the red arrow).

Photosynthesis, and in a narrow sense PSII, responds to incident light in a characteristic manner, which is observed typically in a natural day–night cycle (Fig. 1). Under low-light conditions where light intensity limits photosynthesis, the rate of photosynthetic activity, measured by oxygen evolution in thylakoids for example, increases linearly in proportion to the light intensity. The excitation energy captured by light-harvesting antennae containing Chls and carotenoids is efficiently transferred to the reaction centers of PSI and PSII for photochemical reactions under these conditions. This is the most favorable and healthy condition for chloroplasts, under which they perform photosynthesis smoothly. It should be noted, however, that even weak illumination causes a certain level of photodamage to PSII (Keren et al. 1995, Takahashi and Murata 2008).

With increasing light intensity, the rate of photosynthesis decreases gradually and reaches a plateau where no further increase in photosynthesis is observed. At this stage, PSII is photodamaged, but is also recovered through a repair system, and the rates of damage and repair are balanced. Around this stage, PSII dissipates the excessive light energy as heat to avoid light stress. Energy-dependent quenching (qE) (Briantais et al. 1980) of the well-known non-photochemical quenching of Chl fluorescence (NPQ) (Genty et al. 1989) operates through activation of the xanthophyll cycle (Demmig-Adams 1990) by acidification of the thylakoid lumen. Light-harvesting Chl *a*/*b*–protein complexes (LHCIIs) are the main antenna complexes in higher plants that bind Chl *a* and *b* and xanthophylls (Standfuss et al. 2005). Reversible aggregation of trimers of LHCII in the grana of higher plant chloroplasts (Horton et al. 1991) is believed to be essential for qE. The PsbS protein, a nuclear-encoded PSII subunit, is also suggested to be involved in the photoprotective process (Li et al. 2000, Dominici et al. 2002, Wilk et al. 2013).

When light intensity increases even further, photosynthetic activity declines significantly. At this stage, the rate of photoinhibition prevails over that of protection and repair, and PSII is in a typical photodamaged state in which the reaction center-binding protein D1 of PSII is damaged preferentially and the damaged D1 protein is degraded by specific proteases (Barber and Andersson 1992, Aro et al. 1993). Degradation of the D1 protein requires successive movement of proteins, including detachment of CP43, release of photodamaged D1 protein from the PSII core, and migration of filamentation temperature-sensitive H (FtsH) proteases from the stroma thylakoids to the grana margin (Yamamoto et al. 2008, Yoshioka et al. 2010, Yamamoto et al. 2013). However, protein movement in the thylakoid membrane may be hindered when proteins suffer from photo-oxidative modification by reactive oxygen species (ROS), which results in irreversible aggregation or cross-linking of proteins (Yamamoto 2001, Yamamoto et al. 2008). The so-called bulk lipids, which are mobile and laterally diffuse in the thylakoid membranes, may play an active role in changing the distribution of the PSII/LHCII supercomplexes in the thylakoids. For membrane fluidity, fatty acids of membrane lipids should be polyunsaturated, which inevitably results in lipid peroxidation when the membranes are subject to oxidative stresses such as high light or heat (Yamashita et al. 2008, Chan et al. 2012). In addition, the by-products of lipid peroxidation, i.e. singlet oxygen molecules (^1^O_2_) and carbonyl compounds including malondialdehyde, may cause modification and damage to the nearby proteins (Halliwell and Gutteridge 2007). In the present review, we provide an overview of the processes by which PSII avoids and tolerates light stress, and discuss the role of membrane fluidity in regulation of the movement of PSII/LHCII, which is responsible for photoprotective processes. Irreversible protein aggregation is suggested to threaten the photoprotective system of chloroplast thylakoids.

## Reversible Protein Aggregation Under Moderate Light Stress

Excessive illumination of the thylakoids induces reversible aggregation of LHCII (Horton et al. 1991, Duffy et al. 2013) and irreversible aggregation of PSII core subunits (Yamamoto 2001, Yamamoto et al. 2008). These two processes are independent, and which form of aggregation occurs is determined by the light intensity. Reversible aggregation of LHCII is observed in thylakoids when excessive light energy is dissipated as heat, where the singlet excited state lifetime of Chl *a* decreases through qE of NPQ (Muller et al. 2001, Holt et al. 2004, Johnson et al. 2011).

The biochemical basis of qE of NPQ is well documented through extensive studies over 40 years (Duffy et al. 2013). Non-photochemical quenching comprises three different kinetic phases, namely qE, state transition quenching (qT) and photoinhibitory quenching (qI) (Muller et al. 2001, Holt et al. 2004). The qE component, which dissipates excessive light energy as heat, is dependent on light-induced uptake of H^+^ in the thylakoid lumen by photosynthetic electron transport from water to NADP^+^. Acidification of the thylakoid lumen activates two components necessary for qE. One is violaxanthin de-epoxidase, an enzyme responsible for de-epoxydation of the xanthophyll cycle carotenoid violaxanthin (Yamamoto and Kamite 1972). Through the action of this enzyme, violaxanthin is converted to zeaxanthin. Another component activated by luminal acidification is the PsbS protein (Li et al. 2000, Dominici et al. 2002, Holt et al. 2004). The importance of the pH gradient formed across the thylakoid membrane and acidification of the lumen in the photoprotective responses was confirmed in a recent study of an Arabidopsis mutant lacking the two-pore potassium channel that controls the balance of ΔpH and membrane potential Δψ in the proton motive force (Carraretto et al. 2013). 

**Fig. 2:**
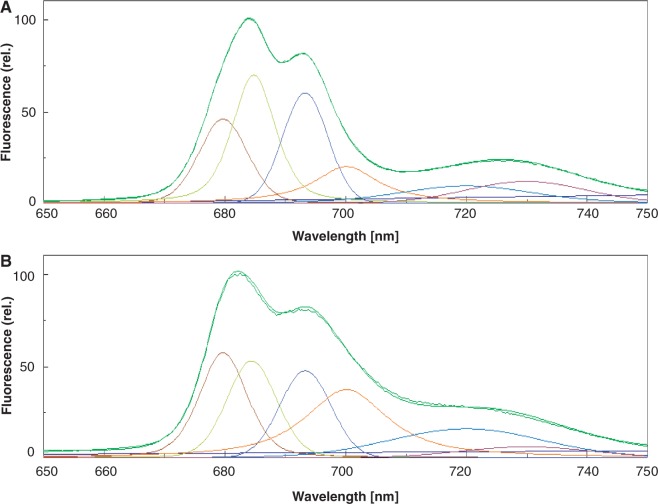
Chl fluorescence emission spectra of spinach thylakoids at 77K. The excitation wavelength was 435 nm and the band width was 20 nm. The emission wavelength was 650–750 nm and the band width was 2.5 nm. The typical fluorescence emission spectra (green curves) are shown with Gaussian decompositions. The curve with little noise is the original fluorescence emission spectrum, while the smooth curve corresponds to the sum of the six Gaussian curves showing the validity of the decomposition results. Six main components were identified in accordance with Stoitchkova et al. (2006) and are referred to as F680 (red curves: peak, 681 nm; half band width, 10.1 nm), F685 (green-yellow curves: peak, 685 nm; half band width, 9.3 nm), F695 (blue-purple curves: peak, 693 nm; half band width, 9.2 nm), F700 (orange curves: peak, 700 nm; half band width, 15.8 nm), F720 (blue curves: peak 720 nm; half band width, 21.9 nm) and F735 (red-purple curves: peak 735 nm; half band width 23.4 nm), respectively. The curves correspond to the fluorescence maxima of the trimeric and monomeric forms of LHCII (F680), the PSII reaction center complex (F685), the core antenna complex of PSII (F695), the aggregated trimers of LHCII (F700), the core complex of PSI (F720) and LHCI (F735). Low emission of PSI fluorescence at the long wavelength is the consequence of a decrease in the quantum efficiency of the photomultiplier used in the fluorescence spectrophotometer. (A) Fluorescence from thylakoids incubated in the dark. (B) Fluorescence from thylakoids illuminated with high light (light intensity: 1,000 µmol photons m^−2^ s^−1^). An increase in the ratio of F700:F680 at high light indicates conversion of free LHCIIs to aggregated LHCIIs.

Aggregation of LHCII associated with qE has been studied with isolated LHCII (Ruban et al. 1994, Ruban and Horton 1999, van Oort et al. 2007). These studies suggest that aggregation of LHCII forms an efficient excitation energy trap. The best and most convenient method for detection of LHCII aggregation is measurement of 77K Chl fluorescence (Stoitchkova et al. 2006, Haferkamp et al. 2010). Aggregates of LHCII show a fluorescence emission peak at 700 nm (Fig. 2). Because aggregation of LHCII formed under moderate light stress is reversible, detection of the aggregates by SDS–PAGE is difficult. In contrast, measurement of 77K Chl fluorescence is simple and provides quantitative data (Horton et al. 1991, Haferkamp et al. 2010, Yamamoto et al. 2013).

Of the other factors involved in NPQ, qT is not prominent in higher plants, and therefore its contribution to NPQ should be relatively small. The qI component is related to photoinhibition and hence irreversible protein aggregation is involved, as will be discussed later.

## Irreversible Protein Aggregation or Protein Cross-Linking Under Severe Light Stress

While the reversible aggregation of LHCII occurs under moderate high light, irreversible protein aggregation of the PSII core subunits is observed typically under extreme high light. Aggregation of the D1 protein is observed in parallel with photoinhibition of PSII, where ^1^O_2_ produced by the acceptor-side photoinhibition of PSII and endogenous cationic radicals such as P680^+^, the oxidized form of the primary electron donor to PSII produced by the donor-side photoinhibition of PSII, are responsible for formation of the protein aggregates (Yamamoto et al. 2008). Three aggregates were identified in spinach thylakoids and PSII membranes: aggregates between D1 and D2 (Ishikawa et al. 1999); D1 and the α-subunit of Cyt*b*_559_ (Barbato et al. 1995, Ishikawa et al. 1999); and D1 and CP43, an antenna Chl-binding protein of PSII (Mori et al. 1995, Yamamoto and Akasaka 1995, Ishikawa et al. 1999). Importantly, aggregation was detected not only in vitro, but also in spinach leaves and cyanobacterial cells, in particular under combined stress conditions such as high light and high temperature (Ohira et al. 2005). Zeaxanthin controls formation of Chl triplets belonging to LHCII and prevents subsequent formation of ^1^O_2_ (Dall’Osto et al. 2012). However, under excessive light, a subunit of LHCII also shows irreversible aggregation (Chan et al. 2012). To detect these protein aggregates, Western blot analysis with specific antibodies against the proteins involved in the aggregation was used. Through these studies, it was suggested that the protein aggregation is useful as a measure of oxidative damage to the proteins (Yamamoto 2001, Yamamoto et al. 2008). These studies also implied that irreversible protein aggregation leads to dysfunction of PSII under light stress. Unless protein aggregation is reversed by an appropriate chaperone system (Rosenzweig et al. 2013), accumulation of these aggregates may be a potential threat to PSII. The machinery to reverse the aggregation of the PSII core subunits is not known.

## Lipid Peroxidation Under Light Stress

Lipids constitute a basic matrix of the thylakoid membrane, interacting directly or indirectly with integral and peripheral proteins of PSII (Mizusawa and Wada 2012). In thylakoids of higher plants, the glyceroglycolipids monogalactosyldiacylglycerol (MGDG), which is a non-bilayer-forming lipid, and digalactosyldiacylglycerol (DGDG), a bilayer-forming lipid, are the major lipids present and constitute 50–60 mol% and 20–30 mol% of total lipids, respectively. An additional glyceroglycolipid, sulfoquinovosyldiacylglycerol, and a glycerophospholipid, phosphatidylglycerol, constitute the minor lipids, comprising 5–10 mol% of total lipids (Dorne et al. 1990). All these lipids exist as either ‘bulk lipids’ comprising the lipid matrix of the thylakoids or ‘bound lipids’ closely associated with specific proteins, and support the functions of Chl–protein complexes and other proteins of the thylakoids. Recent X-ray crystallographic data show that the lipids in and around the PSII complexes are not distributed randomly but are present at fixed locations in the membranes (Loll et al. 2007).

Lipids are unique in generating lipid radicals and secondary products, such as aldehydes and possibly ROS, through lipid peroxidation that takes place under oxidative stress conditions. Lipid peroxidation is the process by which lipids are oxidatively converted to lipid peroxides (LOOHs). The whole process consists of initiation, propagation and termination steps. At the termination step, the concentration of LOOHs increases, and LOOHs interact to form a tetroxide intermediate LOOOOL by a Russel-type reaction (Frankel 1980). Decomposition of the tetroxide leads to formation of LOH and either triplet-excited carbonyl ^3^(L = O)* and molecular oxygen, or ground-state carbonyl (L = O) and ^1^O_2_.

Lipid peroxidation propagates in thylakoids once it is initiated. Production of ROS and its damaging effect on the reaction center-binding D1 protein of PSII is well documented (Yamamoto et al. 2008, Pospisil 2009). It was shown with spinach thylakoids that lipid peroxidation causes damage to the D1 protein under moderate heat stress where ROS are generated and damage the protein (Yamashita et al. 2008). In addition, illumination of spinach thylakoids with excessive light induces lipid peroxidation, which in turn damages the D1 protein and the subunits of LHCII oxidatively by ^1^O_2_ produced through lipid peroxidation (Chan et al. 2012). Lipid peroxidation depends on the presence of polyunsaturated fatty acids in the membrane lipids and hence it is highly conceivable that polyunsaturated fatty acids, which are necessary to maintain membrane fluidity, are inevitably linked to lipid peroxidation under oxidative stresses. Measurement of thermoluminescence bands at high temperature (120–140°C) also showed the occurrence of lipid peroxidation in high-light-treated leaves or leaf discs (Ducruet 2003, Havaux 2003, Havaux et al. 2006, Ducruet and Vass 2009).

## The Grana as a Platform for the Function of PSII/LHCII

Thylakoids of higher plant chloroplasts show a folded sac-like structure. The grana comprise 80% of total thylakoids in vivo (Albertsson 2001). Images of stacked or appressed vesicles (grana) and interconnecting membranes (stroma thylakoids) has been revealed by transmission electron microscopy (TEM) (Mustardy and Garab 2003, Dekker and Boekema 2005). Basic structural models for grana have been constructed using these images, and possible reasons for the presence of grana in the thylakoids of higher plant chloroplasts have been discussed (Trissl and Wilhelm 1993, Arvidsson and Sundby 1999, Albertsson 2001, Allen and Forsberg 2001, Chow et al. 2005).

The mechanism of thylakoid stacking has been studied extensively. Stacking has been suggested to be related to the van der Waals interaction as well as the surface charge density of the thylakoid membranes, which is net negative at neutral pH (Barber and Chow 1979, Barber 1982, Chow et al. 2005). Among four major supermolecular complexes in the thylakoids, PSII/LHCII supercomplexes are abundant in the grana, whereas PSI complexes and ATP synthase complexes are excluded from this area and are localized in the stroma thylakoids (Anderson 1999, Albertsson 2001). The abundance of PSII/LHCII in the grana indicates that LHCII plays an important role in thylakoid stacking.

More sophisticated models of grana have been proposed from studies using new techniques, such as three-dimensional reconstitution of TEM images of chloroplast thylakoid membranes and image analysis of negatively stained samples, and both the function and evolutionary significance of grana have been re-evaluated (Mustardy and Garab 2003, Dekker and Boekema 2005, Mullineaux 2005, Shimoni et al. 2005, Austin and Staehelin 2011, Kouril et al. 2011).

PSII/LHCII supercomplexes are abundant in the grana and frequently show a semi-crystalline array in the grana of dark-adapted leaves (Boekema et al. 2000, Dekker and Boekema 2005), although it was also claimed that PSII complexes in semi-crystalline array account for only a relatively small portion of the PSII complexes even in low light/dark-adapted leaves (Goral et al. 2010, Goral et al. 2011, Johnson et al. 2011). PSII core complexes are composed of dimers of the PSII core. Each PSII core contains the reaction center binding-proteins D1 and D2 and the core antenna complexes CP43 and CP47, as well as other smaller subunits. The core dimer of PSII is surrounded by homo- or heterotrimers of major LHCIIs (Lhcb1–Lhcb3) and monomers of other minor LHCIIs (Lhcb4–Lhcb6) (Dekker and Boekema 2005). Lhcb1 and 2 may be reversibly phosphorylated by illumination (Vener 2007).

## Thylakoid Unstacking and Rearrangement of PSII/LHCII Complexes in the Grana Under Light Stress

Although the grana and stroma thylakoids are spatially segregated from each other, they interact through lateral movement of proteins and lipids between the two membrane domains. The dynamic feature of the thylakoids is necessary for disorganization and reassembly of PSII complexes. For example, protein complexes in the grana diffuse up to several hundred nanometers when they need to reach stroma thylakoids (Kirchhoff 2008). There is, however, no general consensus on the density of proteins in the grana. The grana are shown by electron microscopy to be densely packed with the array of PSII complexes (Dekker and Boekema 2005). If this is true for the grana as a whole, the proteins and plastoquinone molecules in the grana cannot diffuse freely in the thylakoids, and electron transport from PSII to PSI and many other diffusion-dependent processes would be delayed significantly. However, a recent study suggests that the grana regions contain ample areas that are free of large protein complexes (Kouril et al. 2012).

Many earlier studies in vitro showed the occurrence of light-induced structural changes of thylakoids. It was reported recently that thylakoids show irreversible unstacking when exposed to extreme high light or moderate heat stress (Yamashita et al. 2008, Khatoon et al. 2009). The thylakoid unstacking was assayed using digitonin fractionation of the thylakoids (Chow et al. 1980), where heavy grana-enriched pellets were collected by low-speed centrifugation, and the content of Chls in the pellet relative to the the total Chls in the thylakoids was determined. A decrease in Chl content in the pellet indicates a decrease in the amount of grana, which was explained by the authors as unstacking of grana. It was recently shown with spinach leaf discs that a similar change in the amount of the grana occurs when leaves were exposed to extreme high light (unpublished data). Irreversible unstacking of the thylakoids by heat and excessive light was also observed with differential scanning calorimetry and circular dichroism spectroscopy (Dobrikova et al. 2003). A more recent work using small-angle neutron scattering demonstrated that the thylakoid membrane system has the capacity to respond dynamically to the illumination of leaves (Unnep et al. 2014).

**Fig. 3:**
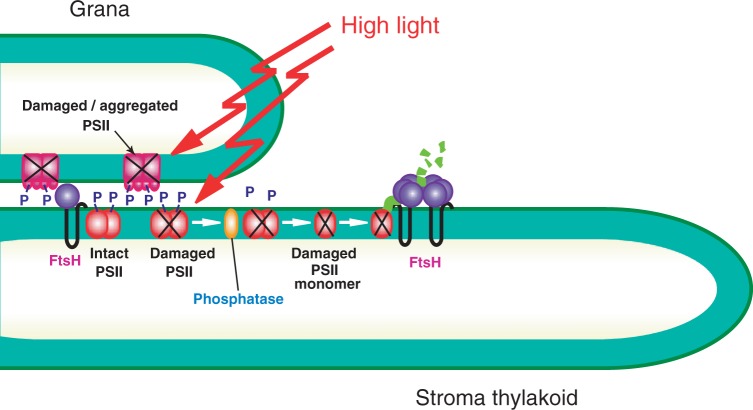
Schematic diagram of the events occurring in the grana and stroma thylakoids under excessive illumination. A horizontal view of the stacked thylakoids is shown. PSII/LHCII supercomplexes (light red) are abundant in the grana regions (LHCII complexes are omitted in this figure). Upon excessive illumination, a portion of the PSII proteins including the reaction center-binding D1 protein is phosphorylated (shown as ‘P’), which protects the proteins from photodamage. However, once PSII complexes, which are present as dimers, are damaged by excessive light (shown by ‘X’), they move to the grana margins, are disintegrated into monomers, and the damaged D1 proteins are dephosphorylated by phosphatases (orange) located in the grana margins. The D1 proteins are then degraded by proteases including FtsH (purple) that are also located in the grana margins. A portion of the photodamaged PSII complexes in the grana (dar red) are immobilized by irreversible protein aggregation. To facilitate degradation of the photodamaged D1 protein in the grana margins, partial unstacking of the thylakoids may be necessary, because it may increase the area of the grana margins and also stimulate mobilization of PSII complexes and the proteases responsible for proteolysis.

Direct observation of thylakoids by electron microscopy supports the above-mentioned biochemical data in showing partial unstacking of spinach thylakoids. More exactly, outward bending of the grana margins occurs under light stress (Yamamoto et al. 2013). Cryo-transmission electron microscopic observation of Arabidopsis leaves showed that the partition gap between adjacent thylakoids is not affected by illumination, but the luminal width of chloroplasts became wider compared with that of dark-adapted leaves. These results may indicate swelling of the thylakoids under illumination (Kirchhoff et al. 2011). If partial unstacking or swelling of the thylakoids occurs, it may prevent possible energy transfer from LHCII in one membrane layer to PSII in the adjacent membrane layer, and the overexcitation of PSII that was hypothesized previously (Mullineaux 2005) should be avoided. Grana stacking induced by addition of salts to the thylakoid suspension stimulates ROS production under illumination (Khatoon et al. 2009). These results suggest that thylakoid unstacking occurs to avoid production of ROS. Partial unstacking and swelling of the thylakoids should also increase the area of the grana margins where photodamaged D1 proteins are removed by proteolytic degradation of the proteins and replaced by new copies (Yamamoto et al. 2013) (Fig. 3).

The factors responsible for light-induced mobilization of PSII/LHCII in the grana are unknown. The outer loop segments of LHCII seem to be important in the lateral reorganization of LHCII and thylakoid stacking (Barzda et al. 1996, Dobrikova et al. 2003). Recently, it was suggested that PsbS controls the association and dissociation of monomeric LHCII and trimeric LHCII (Betterle et al. 2009). Consistent with this suggestion, using an Arabidopsis mutant lacking and overexpressing PsbS, the PsbS protein was shown to change the mobility of Chl–protein complexes within the thylakoid membranes (Goral et al. 2011). However, it remains unknown how the PsbS protein interacts with LHCII. An additional factor involved in mobilization of Chl–proteins is protein phosphorylation. Several protein subunits of PSII/LHCII in higher plant chloroplasts, including D1, D2, CP43, PsbH and LHCII, are reversibly phosphorylated (Bennett 1977, Bennett 1984, Allen 1992, Vener 2007). STN7 and STN8 kinases are responsible for phosphorylation of LHCII and PSII core proteins, respectively (Depege et al. 2003, Bellafiore et al. 2005, Bonardi et al. 2005, Vainonen et al. 2005). The light-induced phosphorylation of PSII proteins induces repulsion between the proteins, which causes detachment of LHCII from the PSII core. This repulsion may be the basis of reorganization of PSII/LHCII complexes in the thylakoids associated with the qE process of NPQ under moderate high light, as well as state transitions under changing light quality. Fluorescence recovery after photobleaching demonstrates strong light-induced mobilization of Chl–protein complexes in the thylakoids of intact spinach chloroplasts (Goral et al. 2010). No such mobilization is seen in Arabidopsis *stn8* and *stn7/stn8* mutants, which suggests that protein phosphorylation is necessary to facilitate the protein mobility. The role of membrane protein phosphorylation in the overall structural changes was studied recently (Fristedt et al. 2009). Protein phosphorylation also causes repulsion between the adjacent thylakoid membrane layers, leading to an increase in the distance between the adjacent thylakoid membranes. Under the same light conditions, the grana diameter decreases. These results may be explained by swelling of the grana thylakoids under excessive illumination. Single-particle tracking, in which a single LHCII complex was labeled with a microsphere and its motion was monitored with a differential interference contrast microscope, demonstrated that phosphorylated LHCII in the stroma thylakoids is more mobile than the non-phosphorylated LHCII (Consoli et al. 2005). Although LHCII movement was probably not monitored in the grana in this experiment, the results suggest the importance of protein phosphorylation in the movement. Recently, experiments were carried out with model membranes composed of the plant lipids MGDG/DGDG and LHCII complexes from dark-adapted spinach leaves (lipid–LHCII) or those from leaves illuminated with high light (lipid–LHCII–HL) (Janik et al. 2013). The lipid–LHCII membranes, in which LHCII complexes are not phosphorylated, assemble into planar multibilayers, whereas the lipid–LHCII–HL membranes, in which LHCII complexes are partially phosphorylated and contain zeaxanthin, form a less ordered structure, implying a positive role for phosphorylation of LHCII in its mobilization in the grana. The effects of high light itself should also be considered here to explain the results.

Recently, an important topic concerning grana structure was reported. The CURVATURE THYLAKOID1 (CURT1) protein family identified in *Arabidopsis thaliana* (Armbruster et al. 2013) is present in the grana margins and regulates grana formation. Lack of the protein caused formation of flat lobe-like thylakoids with fewer grana margins, whereas overexpression of the protein resulted in an increased number of membrane layers in the grana. This protein may be associated with long-term acclimation of plant chloroplasts to changing light conditions, but it will be interesting to know how the CURT1 protein functions in response to different light conditions to form grana.

## Possible Interference of Irreversible Protein Aggregation in the qE Mechanism of NPQ

**Fig. 4:**
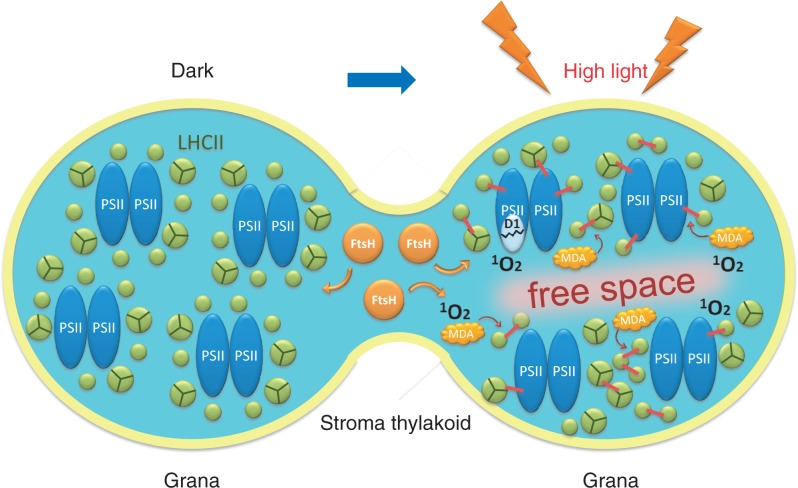
Schematic diagram of events taking place in the grana and stroma thylakoids under excessive illumination. This diagram shows a top view of a thylakoid (light blue), which has a rounded shape and is stacked with other thylakoids to form the grana. Here, two grana-forming thylakoid domains are depicted (left, dark control; right, illuminated with high light), which are interconnected by a stroma thylakoid. PSII core complexes (blue) are surrounded by LHCII complexes (green). When the thylakoids are illuminated with high light, the PSII/LHCII complexes are mobilized, probably by phosphorylation, and LHCII complexes form reversible aggregates to dissipate the excess light energy as heat. Simultaneously, free spaces are generated in the grana and the damaged PSII complexes move to the grana margin regions to react with proteases such as FtsH (orange) using these free spaces. However, when the light intensity is extremely high, ^1^O_2_ molecules are produced in PSII/LHCII complexes through photochemical reaction or in the lipid matrix through lipid peroxidation, and the reactive oxygen species damage the proteins and lipids to form irreversible aggregates. MDA may also participate in this process. These protein and lipid aggregates hinder the movement of PSII/LHCII complexes on the membranes, and the function of the thylakoids may finally deteriorate.

If irreversible protein aggregation or protein cross-linking takes place not only under extreme high light, but also in moderate high light, it may interfere in the qE mechanism of NPQ and prevent efficient dissipation of excessive energy as heat in the latter light condition. Our preliminary results with an ^1^O_2_-sensitive fluorescence probe and 77K Chl fluorescence measurement show that illumination of spinach thylakoids with weak light is enough to induce ^1^O_2_ and irreversible protein aggregation (unpublished data). If ^1^O_2_ is also generated in leaves or cells under weak illumination in vivo, it may indicate that irreversible protein aggregation that takes place in a wide range of light intensities may damage the qE process of NPQ, in which aggregation of LHCII becomes irreversible through oxidation of LHCII by ^1^O_2_ (Fig. 4).

In relation to the effects of high light on PSII, it was recently observed that high-light treatment and moderate heat treatment of spinach thylakoids and PSII membranes induce lipid peroxidation, which in turn leads to irreversible protein aggregation (Chan et al. 2012). Thus, lipid peroxidation may be another oxidative process that interferes in the qE mechanism of NPQ in thylakoids under moderate high light. The physiological significance of irreversible protein aggregation under light stress has long been sought, and it is likely that irreversible protein aggregation disturbs the proper functioning of the qE mechanism of NPQ.

## Concluding Remarks

In the present review, a comprehensive overview of reversible aggregation of LHCII and irreversible aggregation of the PSII core subunits under high light is presented. These processes are independent processes occurring in the grana, and the former is closely associated with photoprotective strategies for PSII, whereas the latter is induced as a result of photoinhibition of PSII. Given that irreversible protein aggregation takes place even under moderate high light, the protein aggregation may hinder photoprotective processes in the thylakoid, in particular the qE of NPQ. Under excessive illumination, lipid peroxidation also occurs and possibly affects membrane fluidity of the thylakoids, but its effect on PSII is still not clear. Important structural changes of the grana, namely swelling and partial unstacking of the grana, may be associated with photoprotection of PSII. Thylakoid dynamics play an important role in the quality control of PSII under light stress. The mobility of the Chl–proteins and other related proteins is essential in the protection of PSII from excessive light and the repair of damaged proteins. Understanding the strategies shown by chloroplasts to avoid disorder and confusion in the crowded grana regions under excessive illumination is crucial to improve the efficiency of photosynthesis in a stressful natural environment with ever-changing light intensity and light quality.

## Funding

This work was supported by the Ministry of Education, Culture, Sports, Science and Technology of Japan [Grants-in-aid for Scientific Research 24570053 Y.Y.].

## Abbreviations

CP43 and CP47: antenna Chl-binding proteins of PSII
D1 and D2: reaction center-binding proteins of PSII
DGDG: digalactosyldiacylglycerol
FtsH: a membrane-bound prokaryotic metalloprotease
LHCII: light-harvesting Chl *a*/*b*–protein complex of PSII
Lhcb1: a major subunit of LHCII
LOOH: lipid peroxide
MGDG: monogalactosyldiacylglycerol
NPQ: non-photochemical quenching of Chl fluorescence
^1^O_2_: singlet oxygen
qE: energy-dependent quenching of Chl fluorescence
ROS: reactive oxygen species
TEM: transmission electron microscopy
